# Exploring Change Mechanisms in a Complex Intervention: Lessons Learned From a Longitudinal Constructivist Grounded Theory Study

**DOI:** 10.1177/23333936251327360

**Published:** 2025-03-20

**Authors:** Stephanie Kipfer, Cedric Mabire, Andrea Koppitz, Sandrine Pihet

**Affiliations:** 1 School of Health Sciences, HES-SO University of Applied Sciences and Arts Western Switzerland – Fribourg, Fribourg, Switzerland; 2 Institute of Higher Education and Research in Healthcare-IUFRS, University of Lausanne, Lausanne University Hospital, Switzerland

**Keywords:** change mechanism, complex intervention, longitudinal qualitative research, constructivist grounded theory, nursing research, implementation science

## Abstract

To develop effective and implementable complex health interventions, we need to understand the mechanisms through which an intervention contributes to desired outcomes. This article provides a practical example of how nursing and other health researchers can qualitatively explore change mechanisms of complex interventions with a longitudinal constructivist grounded theory approach. It shares and discusses lessons learned when applying this approach to explore change mechanisms in the context of a psychoeducational intervention for family dementia caregivers. Important lessons learned included: (1) By comparing data from different time points, changes could be identified even when not explicitly mentioned by participants. (2) Participants exhibited more awareness of changes and related mechanisms in interviews conducted during the intervention compared to those executed after, where they had already integrated the new strategies into their daily lives. (3) Analyses conducted within and across participants for each time point and across all time points revealed time-specific mechanisms, shared patterns and journeys substantially differing from others. (4) Including participants’ quantitative intervention outcomes as additional data in the qualitative analysis helped identify facilitators and barriers to change. In conclusion, our approach produced in-depth knowledge about the intervention’s change mechanisms by considering the complexity and temporal dimensions of participants’ experiences.

## Introduction

To develop effective and implementable complex health interventions, we not only need to know whether an intervention is effective. We also need to understand how an intervention works (Skivington et al., 2021). To improve our understanding in this regard, knowledge of which changes can be expected and the mechanisms contributing to these changes is essential. A mechanism refers to “the processes or events that are responsible for the change” (Kazdin, 2009, p. 419; hereafter referred to as change mechanism). Identifying change mechanisms requires knowing how changes in the outcomes develop, which intervention components contribute to or impede these changes, what intermediate changes occur and what contextual factors may facilitate or hamper changes (Buhse & Mühlhauser, 2015; Kazdin, 2009; Moore et al., 2015a; Moore et al., 2015b; Skivington et al., 2021).

Knowledge about change mechanisms, key intervention components and contextual aspects can help to fine-tune the programme theory (i.e., the theoretical underpinnings of an intervention) and refine the intervention accordingly (Skivington et al., 2021). It can also inform the development of other interventions, particularly when directed at similar populations or promoting similar change mechanisms, and it is highly relevant for the implementation of complex interventions (Kazdin, 2009; Moore et al., 2015a; Moore et al., 2015b; Skivington et al., 2021). More specifically, knowledge about change mechanisms helps optimise change, for example, by adapting the intervention’s content to strengthen or accelerate the change (Kazdin, 2009). Identifying moderators of change (i.e., contextual factors that facilitate or inhibit changes in outcomes) allows clarification regarding which persons benefit the most from the intervention under which conditions (Kazdin, 2007, 2009).

The question arises as to how such essential knowledge about a complex intervention’s change mechanisms can be generated. Two areas of qualitative research relevant to exploring processes illuminate interesting avenues in this regard: (1) constructivist grounded theory (CGT) approaches and (2) qualitative longitudinal research (QLR). CGT approaches are particularly appropriate for exploring social processes in depth, including how, when and why a phenomenon under study changes, as they are oriented towards symbolic interactionism (Charmaz, 2014). Charmaz (2014) defined processes as “unfolding temporal sequences that may have identifiable markers with clear beginnings and endings and benchmarks in between. The temporal sequences are linked in a process and lead to a change” (Charmaz, 2014, p. 17). Furthermore, learning about differences between people experiencing the studied phenomenon and being alert to the conditions under which these differences arise is a priority of the CGT approach (Charmaz, 2014). Therefore, qualitative approaches, such as CGT, are appropriate to gain in-depth knowledge about how intervention participants experience change processes, including how changes in outcomes emerge, what participants do to achieve these changes, or which participants change more or less than others and related aspects (Kazdin, 2007).

QLR “refers to prospective or follow-up studies” (SmithBattle et al., 2018, p. 1) using qualitative methods and two or more data collection time points “to capture and enhance understandings of time perspective and/or change over time” (Nevedal et al., 2019, p. e791). With its focus on time and/or change, QLR is particularly relevant for exploring change processes and mechanisms (Henwood & Shirani, 2022; Neale, 2016). It offers the possibility to deeply examine how subjective experiences and changes related to health behaviours and transitions evolve across time as well as related key time points, patterns, facilitators or barriers to change (Fadyl et al., 2017; SmithBattle et al., 2018; Tuthill et al., 2020). SmithBattle et al. (2018, p. 8) recommended applying QLR “when a long-term perspective on health, illness and the life course will improve clinical care and patient outcomes”, which is highly relevant to nursing. QLR has the potential to increase the knowledge regarding resources and challenges related to coping with health and illness, modifiable factors in these contexts, and social and behavioural outcomes relevant to clinicians and researchers in the nursing field (Bidart, 2013; SmithBattle et al., 2018).

Whereas QLR is common in social sciences, it rarely appears in the nursing literature, where there is currently scarce theoretical, methodological and analytical guidance and practical examples regarding how to conduct robust QLR (Fadyl et al., 2017; SmithBattle et al., 2018; Tuthill et al., 2020). SmithBattle et al. (2018) performed a first methodological review of QLR studies in nursing and found that the methodological quality varied “tremendously” (p. 8), with many studies not meeting general reporting standards for qualitative research. They formulated recommendations for enhancing the quality and reporting of QLR. Tuthill et al. (2020) provided a methodological resource for conducting QLR for nursing and health behaviour researchers. Two method studies on QLR (Audulv et al., 2022, 2023) also made important recommendations about how to conduct QLR studies in health research and how to present the findings of such studies. These are important contributions to the emerging literature on QLR in the field of nursing and health research.

The current paper aims to provide a practical example of how a CGT approach dfwas combined with QLR (hereafter called longitudinal CGT) to explore change mechanisms of a psychoeducational intervention for family dementia caregivers. Based on this exemplary study, we will describe how the approach was practically applied. Moreover, the paper presents and discusses challenges and lessons learned from applying the approach while evaluating a complex intervention. Challenges and lessons learned in the exemplary study shall inform discussions about how to qualitatively explore change mechanisms in the context of complex interventions.

The Background section will provide relevant contextual information about the exemplary study, such as the exemplary study’s aim and rationale, as well as a brief description of the psychoeducational intervention. This will be followed by a description of how the longitudinal CGT approach was applied, focusing particularly on how longitudinal data was collected and analysed. Finally, challenges and lessons learned are presented and discussed.

### Background

The exploration of the change mechanisms with a longitudinal CGT study was performed alongside and embedded in a larger study evaluating a psychoeducational intervention for family caregivers caring for a community-dwelling person living with dementia, called ‘Learning to feel better. . . and help better’ (LFBHB). This psychoeducational group programme aims to empower family caregivers by enhancing their skills to cope with the daily stress of dementia caregiving. In seven 3 hour sessions, caregivers receive active skills training on how to choose and apply appropriate coping strategies (i.e., problem solving, reframing, support seeking) as well as information on dementia, its consequences in relation to the communication and behaviour of the person affected, and care and communication approaches. The intervention’s group format also provides caregivers with the opportunity to share and discuss their experiences with other caregivers and the professional group leader (for more information on the intervention, see Pihet et al., 2024). The original intervention, developed in Canada (Hebert et al., 2003; Lévesque et al., 2002), has been adapted and tested in French-speaking Switzerland (Pihet et al., 2024; Pihet & Kipfer, 2018), yielding positive results for subjective burden, psychological distress, self-efficacy and distress related to the behaviour problems of the person with dementia. The qualitative analyses of these foregoing evaluation studies in Switzerland indicated relationship quality to be an important outcome to explore to better understand the mechanisms leading to the changes observed in the participating caregivers.

Thus, the aim of the exemplary study was to explore changes and related change mechanisms, focusing on the quality of the relationship between caregivers and their family members living with dementia (Kipfer et al., 2024). Changes in relationship quality and related change mechanisms were explored from the perspectives of the caregivers participating in the LFBHB intervention. Kipfer et al. (2024) explored changes in relationship quality across time (namely before, during and after participation in the intervention; see the design and methods section for more detail) as well as change mechanisms, key intervention components and contextual aspects (namely, facilitators and barriers related to the changes). Relationship quality was studied as an outcome that had not yet been addressed in the context of the LFBHB intervention despite its relevance for the quality of life and well-being of the persons with dementia and their family caregivers. By exploring relationship quality, the exemplary study aimed to address the remaining key uncertainties to refine the programme theory and the intervention, three core elements of the new Medical Research Council (MRC) framework for developing and evaluating complex interventions (Skivington et al., 2021). The exemplary study yielded the model on Sustaining Relationship Quality in Dementia (SRQD), which illustrates the strategies that family dementia caregivers developed and applied to maintain reciprocity and mutuality with their family members with dementia (Kipfer et al., 2024). The model also indicates which intervention components facilitated these processes and provides insight into facilitators and barriers to developing and applying supportive strategies. The design and methods used to develop the SRQD model will be described in detail below.

## The Design and Methods of the Exemplary Study

We combined a CGT approach based on Charmaz (2014) with QLR to explore in depth changes in the outcome relationship quality and associated change mechanisms. With this approach, we explored whether, when, how and why relationship quality changed in the context of the LFBHB intervention. The following methods section describes the data collection and analysis of the exemplary study. We focus on design features or methods that are relevant to understanding how we practically applied the longitudinal CGT approach and on specific elements applied to explore changes and related mechanisms in the context of our complex intervention.

### Data Collection of the Exemplary Study

**Figure 1.**
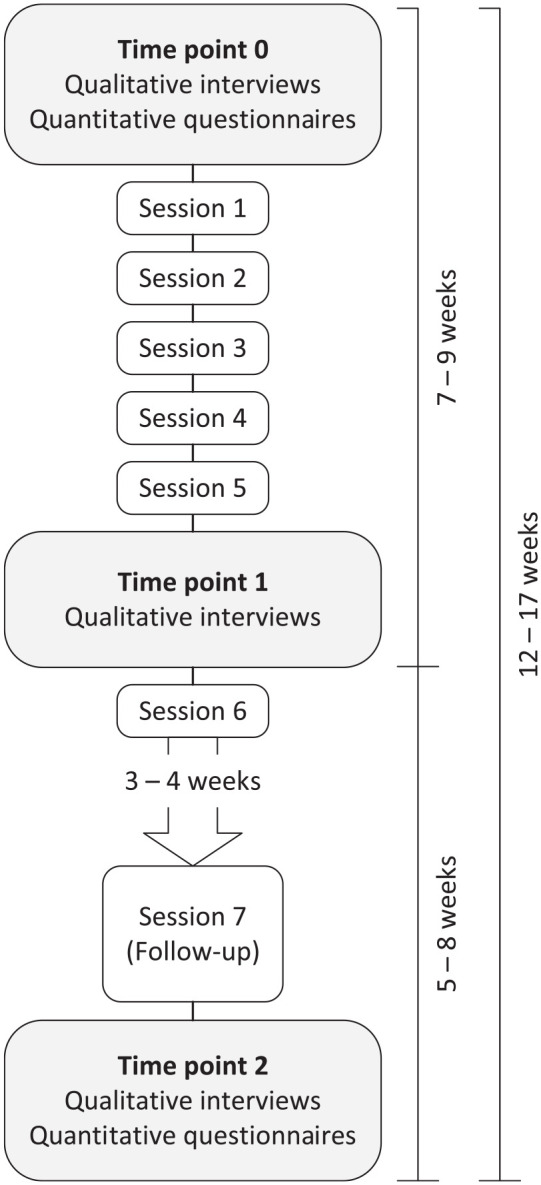
Overview of the data collection process for the exemplary study.

Figure 1 provides an overview of the exemplary study’s data collection process. Longitudinal data were collected with three semi-structured interviews conducted with caregivers before, during and after the intervention. The first and third interviews were executed 1 to 3 weeks before and after participation in the intervention. As situations in the context of dementia caregiving can change quickly, these interviews were performed as close as possible to the start and the end of the intervention to facilitate capturing changes related to the intervention. The second interview was performed during the intervention, namely after session five, when participants had been introduced to all coping strategies taught during the intervention. Overall, interviews were performed in a time frame ranging from 12 to 17 weeks. The second and third interviews were conducted 5 to 8 weeks apart, ensuring that participants had sufficient time to practice and implement new strategies. Between intervention sessions six and seven, participants had 3 to 4 weeks to practice the different coping strategies in their daily lives.

The data collection and analysis were performed simultaneously; thus, interview questions were continuously adapted to gather rich data on new emerging relevant themes related to different caregivers and time points (Charmaz, 2014). However, to compare different time points and identify changes, all interviews started with the same two questions at each time point, as recommended in QLR literature (e.g., Kneck & Audulv, 2019; Nevedal et al., 2019). Caregivers were asked to describe their relationship with their family member with dementia and how they perceived their situation as a dyad. These initial questions were followed by questions about what happened between the interview time points and additional questions prompted by insights from earlier interviews with the same participant or other caregivers. These were, for example, questions about the importance of physical closeness or possibilities of laughing together. On an individual level, this approach was used to explore and follow the individual processes for each caregiver. The interview guide used in the exemplary study can be found in Kipfer et al. (2024).

Demographic and quantitative data regarding intervention outcomes, collected within the larger study evaluating the intervention, were used as additional information in the qualitative analysis to gain broader insight into change processes. Demographic data (e.g., age, gender, relationship to the person with dementia, living situation) were collected in the interviews before the intervention. Quantitative data on five intervention outcomes were collected before and after the intervention. These outcomes were as follows: caregivers’ subjective burden, psychological distress and self-efficacy, as well as the memory and behavioural problems of the family member with dementia and the caregivers’ associated distress. More information on the demographic data and the quantitative intervention outcomes is provided in Kipfer et al. (2024).

All 13 family caregivers from three separate intervention groups (performed at different time points between Autumn 2020 and Spring 2021) participated in the three consecutive interviews. None of the participants dropped out during the study. Thus, the final sample consisted of 39 interviews and 13 individual journeys. The three intervention groups were performed during the second and third waves of the COVID-19 pandemic. All interviews were performed by a female researcher (SK) with well-developed competencies to perform qualitative interviews and conduct CGT as well as with comprehensive experience working as a nurse with people with dementia and their family caregivers. The interviewer was not involved in providing the intervention. The exemplary study was approved by the local ethics review board of the canton of Vaud, Switzerland (Commission cantonale d’éthique de la recherche sur l’être humain [CER-VD], protocol no. 175/14; ISRCTN13512408). Written and oral informed consent was obtained from all participants before the data collection process. Kipfer et al. (2024) provide a detailed description of the sample and relevant information regarding the ethics approval.

### Data Analysis of the Exemplary Study

The data analysis started after the first interview and continued throughout the entire data collection phase. It followed the steps described by Charmaz (2014), including initial coding and focused coding. Initial coding was performed in each interview with participants from the first group at all three time points. All 39 interviews were coded line by line. Initial codes were created following the principles of Charmaz (2014), such as remaining open and close to the data and reflecting action; for example, strategies developed and applied by caregivers in daily life to stay connected to the family member with dementia. Initial coding was used to order data and recognise processes and gaps in the data that required further exploration. Each time point and group were coded subsequently, and insights from these different steps were integrated into the following analyses. With each new time point, intervention group and analysis step, we gradually shifted from initial coding to focused coding. Focused codes were created by combining and/or regrouping numerous initial codes to create more conceptually focused codes that capture relevant themes, patterns or implied meanings on a more theoretical level. With focused coding, we generally synthesised the codes with the most analytic sense, developed categories and, finally, sorted, synthesised and organised the complete data set. All codes were inductively developed based on the qualitative data. No pre-defined themes or codes were used. The grounded theory method of constant comparison was used to compare and explore similarities and differences between data, codes and categories related to the same and different caregivers, different time points and different groups to progressively develop more abstract constructs (Charmaz, 2014). Insights gained from comparing different entities, including questions, thoughts and assumptions which arose during the analysis process, were continuously documented in memos. Memo writing – a typical grounded theory method – was applied throughout the analysis process to further develop ideas, to inform further data collection and analysis steps and to promote reflexivity (Charmaz, 2014). The coding process aimed to depict similarities as well as the variability in the experiences of the different caregivers to provide a broad picture of the studied phenomenon. The coding system accounted for the main themes and subthemes emerging from the data and their timely appearance in the study. Theoretical coding was applied to clarify how the focused codes and categories are related to each other to move towards a theoretical model, which tells the analytic story on a more abstract level (Charmaz, 2014).

Coding was performed by the first author (SK) who also conducted the qualitative interviews with the caregivers. A second female coder with extensive knowledge in developing, evaluating and providing the LFBHB intervention as a researcher and psychologist (SP) assessed a random sample of excerpts with the developed coding system (Polit & Beck, 2021). Ambiguities and differences were discussed, and naming and definitions were adapted accordingly. Regular peer debriefings with health professionals and researchers from different disciplines were organised to discuss the plausibility of codes; code groups and categories; and their relations, with the aim to reduce interpretation errors and increase the reliability and credibility of the findings (Polit & Beck, 2021). Analytical memos were created throughout the code and category development process, contributing to a research diary that documented each analysis step and the decisions taken along the analysis process. In addition, the analysis process has been translated into graphs to retrace the analysis’ progress and analytical decisions. Atlas.ti version 23 was used to aid the analysis process.

**Figure 2.**
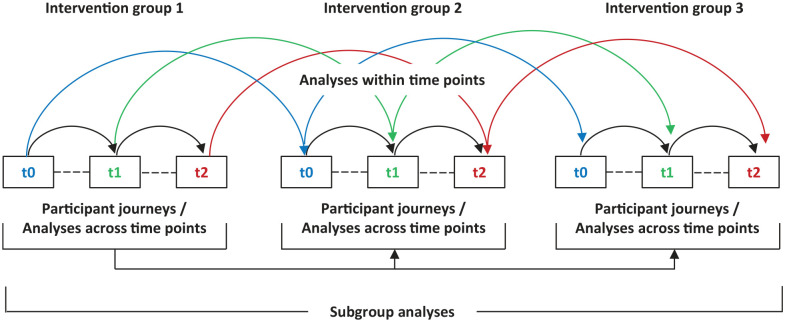
Overview of analyses within and across participants and times.

The analysis was performed according to a data analysis plan developed prior to data collection. Throughout the entire analysis phase, this plan was continuously evaluated and discussed in the research team’s regular meetings. Figure 2 illustrates the different analyses performed. Data were analysed for different data units and on different levels, including within and across time points as well as within and across participants. To perform these different analyses, we examined the data several times with different focusses and perspectives. In terms of the different time points, data were analysed within each time point and compared to the data collected in the other two time points (i.e., before, during and after the intervention). This was performed for each intervention group separately and for the whole sample (three intervention groups together). This was an important first step in identifying relevant themes for each separate time point, which then allowed us to identify themes relevant for all time points and the evolution of these themes through time. A within-participant analysis was performed for each caregiver, using their interview data from all three time points, to explore their individual journeys through time. We particularly assessed changes and stable patterns regarding relationship quality and relevant strategies developed before, during and after the intervention. To do so, all three interview transcripts of each caregiver were analysed a second time in chronological order to create an overview documenting their individual journeys.

In the next step, data from specific caregiver subgroups were compared to obtain supplementary in-depth data regarding differences and similarities between different types of caregivers. The specific subgroups were determined by (1) relevant aspects identified in the preceding analyses, (2) demographic data and (3) quantitative intervention outcomes. Concerning (1), qualitative data were compared for caregivers differing regarding the described prior relationship quality and observations and statements related to their process of grief, dispositional coping styles and health status. Based on (2), data were compared for different relationship types (spouse vs. adult child caregivers), caregiver age (advanced age [>80] vs. younger caregivers) and type of dementia diagnosis of the person with dementia. Concerning (3), data on quantitative intervention outcomes were used to build groups of participants who did or did not improve or deteriorate in these outcomes (i.e., subjective burden, psychological distress, self-efficacy, memory and behaviour problems in the person with dementia, and related distress in caregivers) and to compare their qualitative data regarding relationship quality. Data on these outcomes were integrated into the qualitative analysis after analysing the qualitative data of all three time points of a complete intervention group. The quantitative data was then used as additional information to further analyse the individual journey of each participant. After the analysis of all qualitative data and timepoints from all three intervention groups, we compared the qualitative data of all participants based on their quantitative intervention outcomes.

After analysing all 39 interviews and all different units and levels, the preliminary model was presented by the first and last author (SK, SP) to three health professionals and researchers involved in developing and providing the intervention in Switzerland (i.e., two female psychologists) or Canada (one male psychologist). In the context of a focus group, these persons discussed the relevance and consistency of the model in light of their experiences providing and evaluating the intervention and working with family caregivers. The model’s main components and their relations were validated by the group, whereas some subgroup labels were adapted to be more precise and consistent with the whole model. Subsequently, the first author (SK) presented the adapted model to and discussed it with a group of four former participants (i.e., one female spouse and three male spouses) to assess whether the model resonates with their experiences as intervention participants (Charmaz, 2014). This group validated the main components and provided further insight, deepening the understanding of their links and timing of appearance. This included, for example, precision when they noticed applying new strategies or perceived specific changes or what intervention components were supportive at which time point. By doing so, the discussions with the former participants as well as the one with the professionals contributed to theoretical sampling – a typical grounded theory strategy – that helps gather data to further elaborate and refine categories of the emerging theory (Charmaz, 2014, p. 192).

## Lessons Learned and Discussion

We present here the lessons learned while applying a longitudinal CGT approach to explore changes in relationship quality across time, as well as the associated change mechanisms, key intervention components and contextual factors facilitating or inhibiting such changes in the context of a complex intervention, namely the LFBHB intervention. The lessons learned are based on insights gained while continuously tracking and analysing the research process, particularly by writing reflective analytical memos and by regularly discussing insights and reflections with the research team throughout the entire research process. For each lesson, we will discuss relevant factors that we encountered during the study. These discussions will focus on the benefits and challenges of applying a longitudinal CGT approach when developing, evaluating and implementing a complex intervention. These reflections shall inform future discussions about how to qualitatively explore change processes and change mechanisms in the context of complex interventions and contribute to the emerging literature on QLR in the field of nursing and other health research.

### Longitudinal Data Allow Us to Recognise Changes

Caregivers differed in their reflective skills, affecting their ability to identify and explain changes in relationship quality and the related change mechanisms occurring in themselves and in their interactions with their family members with dementia. Some were able to describe such changes and related change processes directly, whereas for other participants, these were inferred by comparing their interview data from the three time points.

The longitudinal data first allowed us to analyse the perceptions and experiences of caregivers before entering the intervention and to compare these to subsequent time points. Changes in relationship quality could be identified by comparing how caregivers reported on their interactions with their family members with dementia and their consequences in relation to the well-being of the two persons of the dyad at the two consecutive time points. For example, the interviews conducted before the intervention were dominated by the negative changes in relationship quality and well-being caused by dementia. In contrast, in the interviews conducted during the intervention, caregivers mainly reported on how they reflected on their own behaviour and that of the person with dementia and how they were actively trying different coping strategies, thereby gaining more control over their situation. For most caregivers, interviews conducted after the intervention were dominated by their perceptions of positive changes in their relationship quality (e.g., feeling more connected to the family member with dementia) as well as in their well-being and that of the person with dementia. Differences observed between the three interviews provided relevant insights about changes which needed to be further explored in the same or subsequent interviews, even when these changes and related processes were not spontaneously evoked by the participants. This allowed us to investigate what led to these changes and how they further evolved. For example, in t0 interviews, some participants described the disease-related behaviours of their family members as deliberate behaviour that induced negative emotions. In t1 interviews, they described the same behaviour as a symptom of dementia and thus an unintentional behaviour of the family member, helping them to cope with these behaviours. Based on such observations, we, for example, asked participants open questions about how they currently experience and manage specific situations in their daily lives. Participants’ current experiences and practical examples then provided the basis to ask additional questions about what helped them to manage the specific situation in the described way or what made it difficult to manage. Such explorations were also enriched by participants’ statements about what happened between the time points, which helped to identify moments, events or experiences prompting changes in the caregivers’ perceptions and behaviours. Exploring changes across time also allowed us to identify when caregivers started to apply certain strategies in their daily lives. Strategy use could then be analysed in relation to the intervention content that had been provided up to specific time points, which helped to identify key components.

Our findings about the usefulness of the approach to identify changes not explicitly described by the participants or of which they were not fully aware aligns with Thomson’s (2007) claim that QLR allows one to explore differences between what participants say and what they do, which expands traditional qualitative research. When discussing the preliminary model with four former participants, we found further support that the longitudinal CGT approach applied in our exemplary study identified relevant change mechanisms even when these were not explicitly mentioned by caregivers. Indeed, all four former participants recognised their own changes in the outcomes and change mechanisms in the theoretical model that was developed based on all caregiver interviews.

#### Being Open to New Emerging Themes While Controlling for Possible Impacts

The simultaneous data collection and analysis of the CGT approach allowed us to include new emerging themes, warranting further exploration, in the interview guides and questions. Insights from previous interviews and the ongoing analysis were used to refine the subsequent data collection phases, thereby enabling us to continuously extend our knowledge regarding changes and related mechanisms. Indeed, this approach facilitated the acquisition of richer longitudinal data and a broader understanding of the phenomenon under study (Charmaz, 2014; SmithBattle et al., 2018). However, researchers must reflect on how and when to introduce new questions and themes in the interviews to control for a possible impact on participants’ answers, particularly when exploring changes in the context of interventions. In this exemplary study, each interview started with the same two questions before introducing potentially relevant themes that could prompt certain statements in participants. By doing so, answers to the first two questions could be compared between the different time points and changes identified (Nevedal et al., 2019). The use of the same broad questions in each interview has also been described by Kneck and Audulv (2019) as being critical to assessing changes and as a possibility to avoid perceptions of change being prompted or misinterpreted by the researcher. After such initial questions, interviewers can add questions about the time between the data collection time points and relevant aspects observed in earlier interviews with the same participant or in interviews with other caregivers. Researchers need to find a means to refer to themes reported in earlier interviews but simultaneously need to be open to new emerging themes and to different or evolved views of the participant on earlier themes (Kneck & Audulv, 2019). Adapting the data collection to new emerging themes allows one to consider that participants’ perceptions or views can change over time and to focus more on the subjective meaning that participants attribute to their experiences (Audulv et al., 2022).

The possibility to establish a close and trustful relationship with research participants through engaging prolongedly and following the individual journeys is a strength of a longitudinal qualitative approach (SmithBattle et al., 2018). In the exemplary study, all interviews were performed by the same professional at each time point, which can facilitate close and trustful relationships and help to retain participants in the study over time (Tuthill et al., 2020). Building close and trustful relationships was particularly important when exploring relationship quality, as it could involve discussing intimate themes. Aside from the benefits of a prolonged engagement, researchers also must consider the possibility that a prolonged relationship and multiple interviews can impact participants’ experiences, particularly when evaluating interventions (Tuthill et al., 2020).

### Caregivers Are Most Aware of the Change Process While It Is Occurring

Interviews conducted during and after the intervention differed substantially in relation to the depth and comprehensiveness of information about changes in relationship quality, related change mechanisms and key intervention components. In the interviews during the intervention, most caregivers precisely described the changes they observed in themselves, their family members with dementia and their dyadic interactions. The changes described referred to their own behaviour, their well-being or that of the person with dementia, as well as to interactions with and feelings towards the family member with dementia. Participants further described what internal and/or external processes precipitated these changes and which intervention components contributed in this regard. They provided examples from their daily lives, such as how they transferred new insights gained during the group sessions to their situation and implemented them, including the consequences of these actions. For example, participants described how they changed their way of thinking to reduce their negative emotions after working on a painful situation in the group with the help of the group leader and other participants.

In contrast, in the interviews after the intervention, caregivers described more general changes focusing primarily on their well-being or that of the person with dementia and more general strategies they applied to facilitate positive interactions with the family member with dementia and to reduce daily life stress for both members of the dyad. Caregivers provided less detailed information and fewer examples of how they changed their behaviour and what facilitated these changes. When asked for examples, many caregivers struggled to recall specific ones. Overall, after the end of the intervention, caregivers appeared to be less aware of their change processes, as they seemed to have already integrated the key intervention components and the related strategies into their daily caregiving lives.

The observed differences regarding awareness and detailedness of reports in terms of changes, related mechanisms and key intervention components illustrate a crucial element of exploring change processes. Collecting data at time points when participants are possibly most aware of them is pivotal in generating knowledge which is essential for nursing and other health clinicians and researchers aiming to develop and implement interventions to support health behaviours in the people they care for (Tuthill et al., 2020). QLR can be particularly helpful in this regard, as it allows the exploration of participants’ journeys “along the way” while changes and related processes unfold (Neale, 2016, p. 119). Compared to a retrospective study, a “longitudinal [prospective] study takes place in the ‘real’ present, in which the individual and his or her context are synchronous” (Bidart, 2013, p. 258). Jerolmack and Khan (2014, p. 178) emphasise that what people say is a poor predictor of what they actually do, as “meaning and action are collectively negotiated and context-dependent”. Therefore, observing participants in their daily lives may provide more accurate data about behaviour. Performing interviews during the intervention enabled the exploration of participants’ experiences in their ‘real presents’ while they were actively guided and thus actively engaging in practicing new strategies and behaviours in their daily lives. Although we did not perform observations, this circumstance may have helped to approximate as closely as possible participants’ actual daily life situations within an interview and thus to increase the richness and accuracy of the data on behaviours and mechanisms.

Collecting data at multiple time points allows to compare diverse ‘presents’ (Bidart, 2013). This is important, as perceptions about earlier experiences can change when caregivers further develop their knowledge, skills and experiences (Bidart, 2013) so that retrospective descriptions may underscore other aspects as important (Bidart, 2013; Fadyl et al., 2017; Kneck & Audulv, 2019). Participants’ reinterpretations, either implicit or explicit, provide significant insights regarding, for example, how earlier events changed participants’ experiences or perceptions (Lewis, 2007). As described by Fadyl et al. (2017), we recognised interpretations at specific time points as well as changes in how participants interpreted their situation as highly valuable data. Analyses per time point enabled us to capture and preserve participants’ interpretations at specific time points, thereby revealing insights into time-specific experiences and processes for each of the three time points. Comparing interpretations of different time points provided valuable insights into potential changes and related mechanisms that could then be further explored. Over time, researchers’ interpretations can also change as they deepen their understanding of individual participants and the phenomenon under study (Lewis, 2007). This requires researchers to be reflective about their own journey and to carefully document shifts in interpretations throughout the research process (Neale, 2016). Overall, managing different interpretations of participants and researchers and their impact on the analysis can be challenging, necessitating special attention during the analysis process (Fadyl et al., 2017; Thomson & Holland, 2003).

#### Defining the Number of Participants and the Number and Timing of Data Collection Points

Studying a phenomenon while it unfolds is a strength of QLR. However, it is also associated with certain methodological challenges, such as deciding when to finish an analysis or estimating how many participants are needed when new themes emerge at new time points and as participants evolve over time (Neale, 2016). It has been recommended for QLR studies to recruit more participants than typically anticipated to compensate for drop-outs and to obtain sufficient data to improve the trustworthiness of the findings (Kneck & Audulv, 2019; Nevedal et al., 2019; Tuthill et al., 2020). However, following this advice may be highly demanding when the different levels of analyses allow multiple sub-analyses. In terms of subgroup analyses, Calman et al. (2013) recommended reflecting on whether there are strong theoretical or practical reasons to perform sub-analyses for certain participant groups. In the exemplary study, certain sub-group analyses were determined by themes that only emerged in the later stages of the analysis while comparing all 13 caregiver journeys. Although this approach yielded essential insights, some patterns require further exploration, as they rely on few caregivers. This observation highlights the importance of carefully considering how additional data could be obtained to further explore emerging themes. When evaluating interventions, such sampling strategies necessitate special attention, as interviewing new participants or reinterviewing certain participants may be challenging in this context.

An important challenge in QLR concerns defining the number and timing of the data collection time points, which obviously depend on the “nature of the illness or condition” under study (SmithBattle et al., 2018, p. 4) as well as on the nature and format of the intervention. Collecting data in more than two time points can help to capture change more comprehensively (Audulv et al., 2023). The three data points (before, during and after the intervention) in the exemplary study provided particularly rich information about changes in outcomes and change mechanisms, important parts of which would have been missed with traditional approaches of conducting qualitative interviews only after the end of the intervention or before and after. Indeed, having at least three data collection time points allows one to “compare intervals, movements and ‘ways of moving”’, facilitating the possibility to explore changes in participants’ perspectives and priorities (Bidart, 2013, p. 263).

Chosen time points should correspond to specific stages or markers of a transition and should also help participants “remember, objectify or correct an imprecise memory or a subjective reconstruction” (Bidart, 2013, p. 258). In the exemplary study, the first and third data collection points were related to the beginning and end of the intervention. Interviews were performed 1 to 3 weeks before and after participation in the intervention to capture the ‘present’ experience as closely as possible to these two key transitions. The second data collection point was placed when participants had been introduced to all components of the intervention, namely after the fifth session. This was based on the hypothesis that some changes in outcomes should by then have been triggered in all participants, even when the most relevant components associated with these changes differed between participants. The analysis revealed that this second time point generated richer and more detailed data regarding change mechanisms and related intervention components compared to the third time point. In this third time point, participants appeared to have already integrated their new knowledge and skills so that the change mechanisms were no longer ‘present’. This suggests that data collected at later follow-ups, such as 6 or 12 months after the intervention, would be less useful to explore which intervention components facilitated changes in the caregivers. Other challenges when selecting appropriate time points are that participants may not all change at the same time (Kazdin, 2007) or that relevant contexts can change during the data collection phase. For example, in the exemplary study, we had to deal with changing sanitary restrictions due to the second and third waves of the COVID-19 pandemic. Most importantly, researchers must consider participants’ resources, especially when performing research with vulnerable populations, such as family caregivers of persons with dementia. They often experience chronic stress and a heavy burden. Researchers must carefully consider how much time and how many data collection points are necessary and organise additional support if appropriate (e.g., a support person for the person with dementia during the interviews).

### Enriching Data Through Multiple Perspectives

The longitudinal approach offered the potential to analyse the data for each individual participant, for all participants or for specific subgroups per time point and across all time points. Analysing the trajectory of each participant enabled us to explore in depth their individual change processes through time (Nevedal et al., 2019). On the other hand, analysing data for all caregivers or for specific subgroups within and across time points allowed us to identify shared patterns for each time point and their evolution across time. Analyses within and across participants and within and across time are important to gain a meaningful and in-depth understanding of the longitudinal data (Devonald & Jones, 2023; Fadyl et al., 2017; Thomson & Holland, 2003; Tuthill et al., 2020).

In their method study on approaches for integrating time and/or change in QLR findings, Audulv et al. (2023) distinguish between data collected and analysed ‘over time’ and ‘through time’. Collecting and analysing data ‘over time’ focusses on each specific time point and on comparing differences between time points, as performed for the three time points in the exemplary study. When collecting and analysing data ‘through time’, one focusses also on what transpired between the time points and thus on the whole sequence, thereby trying to understand the change that is occurring (Audulv et al., 2022, p. 2, 2023, p. 2). In the exemplary study, asking questions about what happened between time points, exploring themes of previous interviews in consecutive time points and analysing participant journeys also contributed to the exploration of change processes through time. Moreover, the exemplary study and the lessons learned highlight the relevance of collecting and combining data over and through time to facilitate the exploration of change mechanisms associated with a complex intervention.

#### Performing Analyses Within and Across Time Points and Participants to Explore Changes, Change Mechanisms and Relevant Participant Factors

Data collected before the intervention allowed us to compare caregivers regarding their initial conditions. These ‘starting conditions’ (e.g., having a distanced relationship prior to the onset of dementia) could then be considered when comparing the data of the consecutive time points and individual journeys to identify participants’ characteristics affecting the changes in outcomes and related aspects. Across-participant analysis at each time point revealed how caregivers perceived their relationship before, during and after the intervention, revealing different focal points. Comparing time-specific focal points facilitated the identification of changes in caregivers and relevant aspects for each time point.

Combining within- and across-participant analyses (i.e., comparing individual journeys with the overall findings from each specific time point and across all time points) allowed us to identify exemplary and particular caregiver journeys. This procedure also revealed which caregivers benefitted more from the intervention and which did so less, thereby providing insights into participant factors potentially influencing their learning processes. These included, for example, participants’ cognitive or emotional resources or their attitudes and beliefs. For instance, compared to the most common caregiver journey, the few participants who were unable to accept their situation showed substantially less readiness to develop and apply new coping strategies. Comparing individual and overall journeys for specific subgroups of caregivers further allowed us to identify whether and when changes occurred for each caregiver, as well as to find shared patterns in this regard for specific subgroups. Comparing different types of caregivers based on relevant aspects identified in the qualitative analyses (e.g., close vs. distanced relationship before the onset of dementia) or sociodemographic characteristics (e.g., spouse vs. adult child) revealed further important patterns and differences between these subgroups. For example, caregivers with a distanced relationship before the onset of dementia showed more difficulties in recognising behaviour as dementia-related and in adapting their reactions accordingly compared to their counterparts who described having a close relationship with their family members with dementia.

Comparing data for three intervention groups performed at different time points was especially important in the context of the COVID-19 pandemic, during which different legal restrictions were in force for the different groups. This comparison revealed, for example, that some caregivers experienced stronger limitations in social or shared activities outside the home as having a negative impact on their well-being and that of the person with dementia. Such observations provide relevant additional insights when analysing and interpreting the interview data. Comparing two mixed groups, including spouse and adult child caregivers, with a group of spouse caregivers only revealed some differences in the topics discussed in the group sessions. For example, sexuality was brought up only in the spouse group. Understanding how an intervention interacts with its context is essential when evaluating interventions (Skivington et al., 2021). Such insights can shed light on contextual elements that must be considered when refining and implementing the intervention.

#### Quantitative Data Help in Exploring Barriers and Facilitators to Change

Including quantitative data in the analysis allowed us to compare qualitative interview data, including individual journeys and identified changes, of participants showing improvements in specific or all quantitative intervention outcomes (e.g., caregiver subjective burden) with those of participants displaying deterioration or no improvements in these outcomes. Qualitative and quantitative data were mostly coherent; however, qualitative data captured a broader range of changes. The few caregivers who did not improve on several quantitative intervention outcomes provided relevant insights into barriers that hampered their learning processes, such as not being ready to accept their family member’s disease or their caregiver role. The journeys of caregivers who improved on quantitative intervention outcomes revealed facilitators, such as having a close relationship before the onset of dementia. Using the quantitative outcomes’ results to guide group comparisons on qualitative data allowed us to consider additional factors that were potentially related to our outcome of interest (i.e., relationship quality). In this regard, combining quantitative and qualitative data produced new insights, particularly regarding facilitators and barriers. Moreover, it helped to identify which caregivers benefitted the most from the intervention as well as which would most likely need additional or a different kind of support.

Overall, assessing similarities and differences between different caregivers and their journeys, including, for example, caregivers who did not show improvements, helped to build categories and clarify their relations, which ultimately helped to develop the theory (Miles et al., 2020). Moreover, identifying unexpected pathways and understanding how expected pathways manifest in participants fosters intervention development by nurturing the theory about the intervention’s change mechanisms (Moore et al., 2014, 2015b). Furthermore, comparing the data of individual caregivers, different caregiver subgroups, different intervention groups performed at different time points or data collected at different time points allowed various forms of triangulation. Triangulation can help to gain a broader picture and to understand a phenomenon more comprehensively (Flick, 2019). In the exemplary study, comparing and combining these different perspectives on the data enriched and validated the data through multiple perspectives on changes and associated change mechanisms and helped to gain a more nuanced and multifaceted understanding of participants’ change processes (Miles et al., 2020).

#### Having an Analysis Plan While Being Open to New Subgroup Analyses

Performing analyses within and across time and participants demands a well-organised analysis plan to manage the complexity and harness the richness of longitudinal qualitative data. Before performing the analyses for different time points and subgroups, we developed an analysis plan by outweighing the benefits and limits of the different steps and ways of ordering and prioritising them. This analysis plan was necessary to systematically perform and document the different steps and to ensure that the different analyses influenced each other in the planned way. For example, we introduced quantitative data after all qualitative data for an intervention group had been analysed to avoid the risk of focusing on observations that confirmed the results of the quantitative intervention outcomes (Moore et al., 2015b). This procedure also provided an additional validation of the findings from the first purely qualitative analysis. On the other hand, our plan needed to be sufficiently flexible to integrate new subgroup analyses indicated by the ongoing analysis (e.g., a subgroup of caregivers showing difficulties in coping with grief).

#### Managing a Large Volume of Data Requires Careful Planning and Documentation

Conducting longitudinal studies and managing and analysing the large volume of data collected in these studies requires a substantial amount of time (Fadyl et al., 2017; Tuthill et al., 2020). This particularly applies to performing analyses for different subgroups and time points as well as when exploring new emerging themes and defining subgroup analyses along the ongoing analysis, as performed in the exemplary study. Such an approach requires us to analyse interviews, quotes, codes and categories several times, focusing on different perspectives. Therefore, resources and data management must be planned carefully (Calman et al., 2013; Neale, 2016; Tuthill et al., 2020). In addition, keeping track is essential and can be achieved by documenting, for each analysis step, the aim, findings, related observations and reflections, for example, about a possible impact on other steps of the analysis (see Fadyl et al., 2017 for examples on tracking the coding and analysis process). Tuthill et al. (2020) recommended also documenting which actions produced meaningful findings and which did not. Generally, a longitudinal analysis requires continuous and precise documentation of the coding history and decisions over time as well as a data analysis plan to manage the different levels and units of analysis and strengthen the trustworthiness of the data (Fadyl et al., 2017; SmithBattle et al., 2018; Tuthill et al., 2020).

Visual formats, such as matrices or graphic displays, are helpful when analysing a large volume of data and/or performing many different analyses, such as for different subgroups or time points. They can help to systematically present information, which helps to analyse and better understand the data (Miles et al., 2020). Based on the advice of Miles et al. (2020) on constructing matrices, we created different matrices, depending on what question we aimed to explore or/and which data we aimed to compare. For example, we created matrices that documented and summarised the journey of each participant across time to compare the different caregiver journeys. Another matrix presented a summary of key aspects and reflections per time point to compare participants’ interview data per time point. We also created a matrix to visually combine the quantitative intervention outcomes of each participant with their qualitative interview data per time point and across all time points based on the matrices described before. In addition, we continuously constructed and/or modified network displays to visualise themes and categories and explore relations between them. In the later stages of the analysis, as part of theoretical coding, we used network displays to map and reflect on change processes and related mechanisms. These network displays then built the basis to develop the final theoretical model.

### Reflections on Exploring Complex Change Mechanisms With a Longitudinal CGT Approach

The longitudinal CGT approach used in the exemplary study allowed us to develop a comprehensive model on changes, related change mechanisms, key intervention components and participants’ factors facilitating or hampering change in the context of a complex intervention. By illustrating change mechanisms and influential participant factors, such theoretical models provide an essential basis to guide an intervention’s further development and implementation, as well as those of other interventions (Kazdin, 2009; Moore et al., 2015b; Skivington et al., 2021).

With its focus on exploring changes, related mechanisms as well as barriers to and facilitators of change, the exemplary study corresponds to study type C3 described by Audulv et al. (2023) in their scoping review of designs in QLR studies. Based on how time or change was presented in the findings, Audulv et al. (2023) defined a typology including three article types and seven subtypes. Subtype C3 articles focus on change processes, namely how and why change occurs (Audulv et al., 2023). As in the exemplary study, the findings often included a model illustrating a change process and/or facilitators or barriers to change. According to Audulv et al. (2023), theoretical models on change such as those developed with type C studies help us better understand change.

Using a longitudinal CGT approach to explore change processes associated with a psychoeducational intervention proved particularly useful in the context of dementia caregiving, which is complex and continuously changing. Indeed, the caregiving situation is affected by the disease’s progress, its impact on the well-being of the person affected and their family, the characteristics of the people involved and their available resources to manage the challenges.

Globally, our findings support the view that traditional qualitative interviews conducted only after the intervention, or before and after, have the risk of missing essential information about change mechanisms (SmithBattle et al., 2018; Tuthill et al., 2020). Indeed, it proved to be essential (1) to perform interviews during the intervention, when changes were occurring in participants, to explore change mechanisms and (2) to have at least three time points to identify changes, particularly those which the participants were not fully aware of or which they did not explicitly describe. The changes identified between time points build a basis to explore related mechanisms and intervention components. Two design features were pivotal to identifying changes in participants: Asking the same two broad questions at the beginning of each interview and constantly comparing data collected at the three time points. Simultaneously collecting and analysing data allows us to adapt interview guides to further explore differences observed between time points as well as relevant themes identified throughout the analysis with additional explorative questions. This approach enables us to gain an in-depth understanding of changes and the related mechanisms and intervention components.

Identifying barriers and facilitators was facilitated by comparing the journeys of individual caregivers or certain sub-groups with the data of all caregivers and including quantitative intervention outcomes in the qualitative analysis. However, some of the barriers or facilitators identified in the exemplary study rely on few participants displaying these patterns. Theoretical sampling – a typical grounded theory strategy – was applied in the exemplary study by adapting interview questions to further explore relevant themes and when discussing the preliminary model with former participants to refine the model and its categories. Theoretical sampling could also have been applied to further explore patterns related to facilitators and barriers that required further exploration. This could have been done by reinterviewing participants or by interviewing new participants. If barriers and facilitators are of particular interest, such additional interviews could allow us to explore more facets of these patterns. Such complementary insights are useful to further elaborate and refine the related categories and their relations, which ultimately helps to develop the theory.

The MRC framework for developing and evaluating complex interventions highlights the importance of identifying the mechanisms contributing to changes as well as the contextual factors “which determine and shape whether and how outcomes are generated” (Skivington et al., 2021, p. 2). Our exemplary study mainly provided insight into factors related to participants’ characteristics. However, external contextual factors are also of interest, particularly those related to the intervention’s delivery or implementation such as the group leaders’ skills, the composition or size of participant groups or the frequency of intervention sessions, which would be essential to explore in future studies. An exploratory qualitative approach, as applied in the exemplary study, represents an important first step to prioritising the contextual aspects to assess with further research, particularly regarding participants’ characteristics. The COVID-19 pandemic brought additional contextual factors into play. The influence of this particular context was evident in the qualitative interviews and was considered when analysing and interpreting the data. However, a more in-depth exploration of the impact of this particular factor was not possible within the exemplary study. Indeed, contextual factors are manifold, and it may not be feasible to explore all of them in-depth within one study.

The approach we applied allowed us to develop a theoretical model about change from the perspectives of the intervention’s participants, providing the knowledge base to orient further quantitative assessment, refine the intervention or allow comparisons with other theoretical models. However, after this important first step, further assessment is required in the case of the exemplary study, as the model developed is based on a limited sample size, particularly for findings regarding subgroup analyses. The drawbacks of the applied approach are that it requires substantial resources in terms of time and competencies to manage its complexity; therefore, it may be more feasible with small samples. Nevertheless, our exemplary study confirmed that it is worth investing these resources to capture the complexity of change mechanisms.

#### Combining CGT and QLR

Globally, QLR generates relevant evidence for nursing practice and science as well as for other health professionals. However, researchers aiming to conduct robust QLR need theoretical, methodological and analytical guidance as well as practical examples. In their scoping review of designs in QLR studies, Audulv et al. (2022, p. 2) summarised that there is still a debate about whether “QLR is a methodological approach in its own or a design element of a particular study within a traditional methodological approach” such as grounded theory: In 17% of the reviewed articles, the authors stated having used a QLR design and following a methodological approach (e.g., case study, phenomenology, grounded theory), while in 21%, they reported conducting a longitudinal study or a longitudinal study within a specific qualitative tradition (e.g., grounded theory; Audulv et al., 2022). According to Audulv et al. (2022), studies with a QLR methodology should fulfil specific criteria, such as aiming to explore change within a phenomenon or change in relation to an outcome and to integrate a longitudinal data collection in their method. Changes related to outcomes were frequently explored in intervention studies by comparing participants regarding differences in outcomes and related aspects leading to these (Audulv et al., 2022), as performed in our exemplary study.

Given that more guidance is needed for QLR, our exemplary study provides insight into combining QLR with the well-developed CGT approach, for which there is sufficient literature about how to rigorously apply it. On the other hand, literature on QLR can provide specific guidance on how to collect, analyse and present longitudinal data and thus make the optimal use of these kinds of data. QLR and CGT are particularly easy to combine, as they typically share the assumption that experiences are co-constructed between participants and researchers (Charmaz, 2014; Tuthill et al., 2020). In addition, the simultaneous data collection and analysis typical of CGT is also commonly performed in QLR (Tuthill et al., 2020). Both QLR and CGT allow researchers sufficient flexibility, facilitating innovative and customised approaches to producing rich data (Audulv et al., 2023; Charmaz, 2014). With its inductive approach, the longitudinal CGT design allowed us to acknowledge the complexity of dementia caregivers’ realities by focusing on their voices and experiences. This in-depth knowledge can complement more abstract and simplified theories about change mechanisms with the perspectives of the people participating in the intervention under study. However, more research and literature are needed to develop such approaches further when applied to the development and evaluation of complex interventions.

## Conclusion

The longitudinal CGT approach was helpful in producing in-depth knowledge about a complex intervention’s change mechanisms, key intervention components and relevant participant factors. The approach allowed considering the complexity and temporal dimensions of participants’ experiences. The current article provides a practical example of how change mechanisms of complex interventions can be explored with a longitudinal CGT approach. This article shall encourage and support nursing clinicians and researchers in filling the current knowledge gaps related to the change mechanisms of many complex health interventions and thereby help to further develop and implement effective interventions.

## Statements and Declarations

**Ethical Approval and Informed Consent Statements:** The exemplary study was approved by the local ethics review board of the canton of Vaud, Switzerland (Commission cantonale d’éthique de la recherche sur l’être humain [CER-VD], protocol no. 175/14; ISRCTN13512408). Written and oral informed consent was obtained from all participants before the data collection process.

**Consent to Participate:** Not applicable.

**Consent for Publication:** Not applicable.

**Data Availability Statement:** Data sharing is not applicable to this article, as no data sets were generated or analysed for the current article. The datasets generated and/or analysed during the exemplary study are available from the corresponding author upon reasonable request.
